# Development of a Sensitive Real-Time Fast-qPCR Based on SYBR^®^ Green for Detection and Quantification of Chicken Parvovirus (ChPV)

**DOI:** 10.3390/vetsci5030069

**Published:** 2018-07-25

**Authors:** Luis F. Nuñez, Silvana H. Santander-Parra, Lucas Chaible, David I. De la Torre, Marcos R. Buim, Alexandre Murakami, Maria Lucia Zaidan Dagli, Claudete S. Astolfi-Ferreira, Antonio J. Piantino Ferreira

**Affiliations:** 1 Department of Pathology, School of Veterinary Medicine, University of São Paulo (USP), Av. Prof. Dr. Orlando M. Paiva, 87, CEP 05508-270 São Paulo, Brazil; fabiann7@yahoo.es (L.F.N.); silvanahsp@yahoo.com (S.H.S.-P.); chaible@usp.br (L.C.); daviddelatorreduque@gmail.com (D.I.D.l.T.); alexandre.murakami@usp.br (A.M.); mlzdagli@usp.br (M.L.Z.D.); csastolfi@gmail.com (C.S.A.-F.); 2 School of Veterinary Medicine, Central University of Ecuador, Jeronimo Leiton s/n, EC170521 Quito, Ecuador; 3 Biological Institute, Av. Gaspar Ricardo, 1700, CEP 17690-000 Bastos, Brazil; marcosbuim@biologico.sp.gov.br

**Keywords:** chicken, parvovirus, real time, qPCR, SYBR^®^ Green

## Abstract

Many viruses have been associated with runting and stunting syndrome (RSS). These viral infections mainly affect young chickens, causing apathy, depression, ruffled feathers, cloacal pasting, and diarrhea. Chicken Parvovirus (ChPV) is such an infection and has been detected in chickens showing signs of enteric diseases worldwide. Therefore, the present study aims to develop a sensitive real-time fast-qPCR assay based on SYBR^®^ Green for detection and quantification of ChPV. A 561-bp non-structural (NS) gene was amplified and cloned, and a pair of primers was designed based on conserved nucleotide sequences on the NS gene of ChPV, the intercalating DNA reagent SYBR^®^ Green was employed, and the Fast mode of a thermocycler was used. The assay detects 10^9^ to 10^1^ copies of the genome (CG). The limit of detection (LoD) was estimated to five CG, and the limit of quantification (LoQ) was estimated at ten CG. The standard curve efficiency was 101.94%, and the melting curve showed a unique clean peak and a melting temperature of 79.3 °C. The assay was specific to amplify the ChPV NS gene, and no amplification was shown from other viral genomes or in the negative controls. A total of 141 samples were tested using the assay, of which 139 samples were found positive. The highest CG value of ChPV was 5.7 × 10^6^ CG/uL of DNA without apparent clinical signs of enteric disturbance, and 4.6 × 10^6^ CG/uL DNA were detected in chickens with RSS.

## 1. Introduction

Runting-stunting syndrome (RSS) is a pathogen that afflicts young, one-week-old chicks, causing poor feed conversion, stunting, dwarfism, culling and mortality [1,2,3]. Generally, the chicks present with depression, apathy, ruffled feathers, cloacal pasting, and the most characteristic signs, diarrhea and unsteadiness [3,4]. The etiological agents related to RSS remain unknown; however, many viruses have been detected and implicated as causative agents of the disease, including astroviruses, coronaviruses, rotaviruses, reovirus, parvovirus, and others [5,6,7,8,9,10]. Parvovirus is a viral agent that affects many animals, including mammals such as cows, dogs, cats, rats, and bats [11]. Many experimental infections have been carried out using isolated enteric viruses, such as astrovirus, avian nephritis virus (ANV), rotavirus, and Chicken Parvovirus (ChVP), to elucidate the pathogenesis of RSS [6,12,13]. These studies have determined the ChPV might be an etiological agent associated with RSS and could be considered a principal causal agent of this enteric disease [3,14]. Additionally, in Muscovy ducks, ChPV causes Derzsy’s disease [15]. In chickens, it was associated with curvature of the duodenal loop, pancreatic atrophy, and mesenteritis [16], and it has been detected in chickens and turkeys showing signs of enteric diseases and in apparently healthy animals [17,18,19]. ChPV is a non-enveloped, positive-sense, double-stranded DNA virus that was first reported to cause enteric disturbances in 10-day-old chicks [4]. Many other studies have been done, thereafter, to elucidate the role of ChPV in enteric disorders, diarrhea, and other enteric manifestations [3,13]. The fully assembled ChPV genome is 5275 bp in length and is organized in the non-structural (NS) gene that encodes a non-structural protein and contains ORF1 and ORF2, which are proteins involved in viral replication; the VP1 and VP2 genes that encode the structural proteins that make up the viral capsid, and the NP1 gene that contains ORF3, which is located between ORF1 and ORF2 [20]. Based on the sequences of these genes, a PCR end-point and real-time PCR (qPCR) assays were developed for ChPV identification [21]. The majority of these assays focused on the NS gene [8,22,23] because it is the most conserved gene in ChPV. The first PCR assay developed was described by Zsak et al. [8] where the primers flanked a conserved region of the NS gene, and currently, two qPCR assays were developed based on the same NS region [22]. Another qPCR assay was developed to amplify part of the VP2 gene region [21]. All of these assays showed a high sensitivity for ChPV detection and quantification. In the Brazilian chicken flocks in the recent years, many outbreaks of RSS have been reported where ChPV has been detected, and the NS gene was sequenced, confirming that the sequences belonged to ChPV, identifying the virus in young and old chickens as early as the first day of infection [9,16,24,25,26]. Enteric diseases cause economic losses in poultry, the depreciation of poultry flocks, and increased susceptibility to other diseases, so a rapid, efficacious, affordable, reliable assay should be standardized for the diagnosis of the etiological agent [27,28,29].

In the present study, we report the development of a highly sensitive real-time fast-qPCR assay based on SYBR^®^ Green for detection and quantification of ChPV, which is faster than other current assays due its combination of cycle steps, faster changes in temperature, faster enzymes, and is so sensitive that very low concentrations of the virus present in the enteric contents of animals with or without clinical signs of enteric disease can be detected.

## 2. Material and Methods

### 2.1. Sampling

The sampling comprised 141 samples of enteric content of randomly selected chickens (broilers, laying hens, and breeders) from several states of Brazil and was received between the years 2000 to 2016. The birds were healthy or had history of signs of enteric disease, principally the presence of diarrhea. The age of the birds varied from one-day to several weeks-old. The selected samples were screened for enteric viruses by molecular analysis using real-time PCR (qPCR) for the detection and quantification of ChPV, allowing the qPCR assay to be standardized and validated.

### 2.2. Preparation of Samples and DNA Extraction

A 1:1 suspension was made by placing a portion of a macerated sample of the enteric content into a 2 mL microcentrifuge microtube in 1000 µL of 0.1 M phosphate buffered saline (PBS) pH 7.2, homogenized, and centrifuged at 12,000× *g* for 20 min at 4 °C. A 200 µL aliquot of the supernatant from the suspension was used for DNA extraction with TRIzol reagent (Thermo Fisher Scientific, Carlsbad, CA, USA) reagent according to the manufacturer’s instructions.

### 2.3. PCR

The DNA of the sample used for ChPV NS gene cloning was subjected to PCR amplification using the conditions previously described [8] with some modifications, and with one pair of primers described in Table 1. A PCR reaction used 23 µL of reaction mixture with 0.5 µM of the forward and reverse primers (Table 1), 2× Buffer, 5 mM of each dNTP, 37.5 mM of Mg, and 1 U of Platinum Taq DNA polymerase (Thermo Fisher Scientific, Carlsbad, CA, USA), and 2 µL of DNA. The samples were subjected to PCR amplification under the following conditions: one cycle of 95 °C for 5 min to completely denature the DNA; 35 cycles of 95 °C for 30 s for template denaturation, 56 °C for 30 s for primer annealing, and 72 °C for 45 s for extension; and a final extension step at 72 °C for 10 min. The reaction was maintained at 4 °C until stored at −20 °C. The PCR products were subjected to electrophoresis in a 1.5% agarose gel. The samples were stained with SYBR^®^ Safe DNA gel stain (Thermo Fisher Scientific) and compared with a 100-bp molecular ladder (Thermo Fisher Scientific). The gels were analyzed on a transilluminator and photographed using an Alpha Imager Mini Analysis System (Alpha Innotech-ProteinSimple, San Jose, CA, USA).

### 2.4. Primer Design and Standard DNA Construction

One pair of primers was (PVA-F and PVA-R; Table 1) designed based on the conserved region of the NS gene. To this end, one sample known to be positive for ChPV was subjected to PCR [8] and is described above, amplifying a region of the NS gene. The PCR product obtained was cloned with a pCR 2.1-TOPO^®^TA Cloning Kit (Thermo Fisher Scientific) according to the manufacturer’s instructions. A plasmid was extracted from the transformed *E. coli* cells using PureLink™ Quick Plasmid Miniprep Kit (Thermo Fisher Scientific) according to the manufacturer’s instructions. Three extracted plasmids were sequenced using the M13 forward and reverse primers, and the sequences were edited and analyzed to obtain unique consensus sequences that were analyzed using the BLAST tool to find high similarity with other ChPV sequences present in GenBank. The primer was designed based on alignment with the reference sequence NC_024452.1 ABU-1 from Hungary, and nine other complete ChPV genomes from China, Korea, and the United States present in GenBank and the recombinant plasmid sequence with the cloned segment of the ChPV NS gene, using the software package Geneious version 10.2.3 (Biomatters Ltd., Auckland, New Zealand). The primer sequences are shown in Table 1.

### 2.5. Real-Time PCR—qPCR Assay

The qPCR reaction used 19 µL of reaction mixture that contained 2X of Fast SYBR^®^ Green Master Mix (Thermo Fisher Scientific), 0.5 µM of each primer, 5 µL of UltraPure DNase/RNase-Free Distilled Water (Thermo Fisher Scientific) and 1 µL of extracted DNA. qPCR amplification was performed in the fast mode under the following conditions: one cycle of 95 °C for 20 s to completely denature the DNA, 40 cycles of 95 °C for 3 s for template denaturation, and 60 °C for 30 s for annealing and extension. A dissociation curve (melting) was performed by a gradual increase in temperature (0.3 °C). The qPCR reactions were carried out in a qPCR thermocycler Step One Plus (Thermo Fisher Scientific). All samples were tested in duplicate, and absolute quantification was performed relative to the standard curve used in each run. A negative control was used in all amplifications. The primers targeted a highly conserved region of the NS gene. The flanking region is in the position 2127 to 2222 in the ChPV genome ABU1, and a 96-bp product was amplified. No dimer products were seen in any run.

### 2.6. Determination of Standard Curve for qPCR Assay

A standard curve was constructed using an extracted plasmid that contained the NS gene region cloned as described above; the plasmid was quantified using a NANO Drop (Thermo Fisher Scientific). The web tool DNA Copy Number Calculator was used to calculate the quantity of recombinant DNA plasmid necessary to make a first dilution with a known quantity of DNA copies, then tenfold dilutions were prepared to determine the sensitivity and amplification efficiency of the qPCR assay.

### 2.7. Specificity and Sensitivity of qPCR Assay

A specificity assay using DNA extracted from isolates of FAdV-1, poxvirus, LTI, and fowl adenovirus II vaccines was performed. The sensitivity assay used a standard curve constructed with ChPV recombinant DNA plasmids that were run in qPCR.

### 2.8. Limit of Detection and Quantification

The limits of detection and quantification were determined using the standard curve. The limit of detection (LoD) was defined as the lowest DNA plasmid concentration present in the tenfold dilution series detected by the assay, and the limit of quantification (LoQ) was determined by the lowest DNA plasmid concentration that the assay could quantify and maintain in the linear portion of the standard curve.

### 2.9. DNA Sequencing and Phylogenetic Analysis

From the positive tested samples, ten were randomly selected, and a 561-bp fragment of the ChPV NS gene was amplified using the conventional PCR described above. PCR products were purified using a CleanSweep PCR Purification (Thermo Fisher Scientific) as described by the manufacturer. Each purified product was sequenced in the forward and reverse sense using a BigDye^®^ Terminator v3.1 Cycle Sequencing Kit (Thermo Fisher Scientific). The obtained sequences were edited using the Geneious software package version 10.2.3 (Biomatters Ltd., Auckland, New Zealand) and analyzed using BLAST to determine if the sequences were similar to other ChPV sequences deposited in GenBank. The sequences obtained were aligned with other ChPV sequences from many other countries using the ClustalX2 2.1 software package, and the similarity matrix of nucleotides, amino acids, and the phylogenetic tree was inferred in the MEGA 7 software package [30]. The phylogenetic analyses methods were chosen using the option Find Best DNA/Protein models (ML).

### 2.10. GenBank Accession Numbers

The nucleotide sequences of a portion of the ChPV NS genes identified in the present study were deposited in GenBank under the accession numbers USP 162 (MH176305); USP 238-1 (MH176307); USP 242-62 (MH176311); USP 259-10 (MH176306); USP 259-12 (MH176314); USP 336-7 (MH176309); USP 336-15 (MH176308); USP 399-1 (MH176310); USP 400-1B (MH176313); USP 400-7 (MH176312).

## 3. Results

The primers targeted a highly conserved region of the NS gene. The flanking region is in the position 2127 to 2222 in the ChPV genome ABU1, and a 96-bp product was amplified. No dimer products were seen in any run.

### 3.1. Determination of Standard Curve

**Figure 1:**
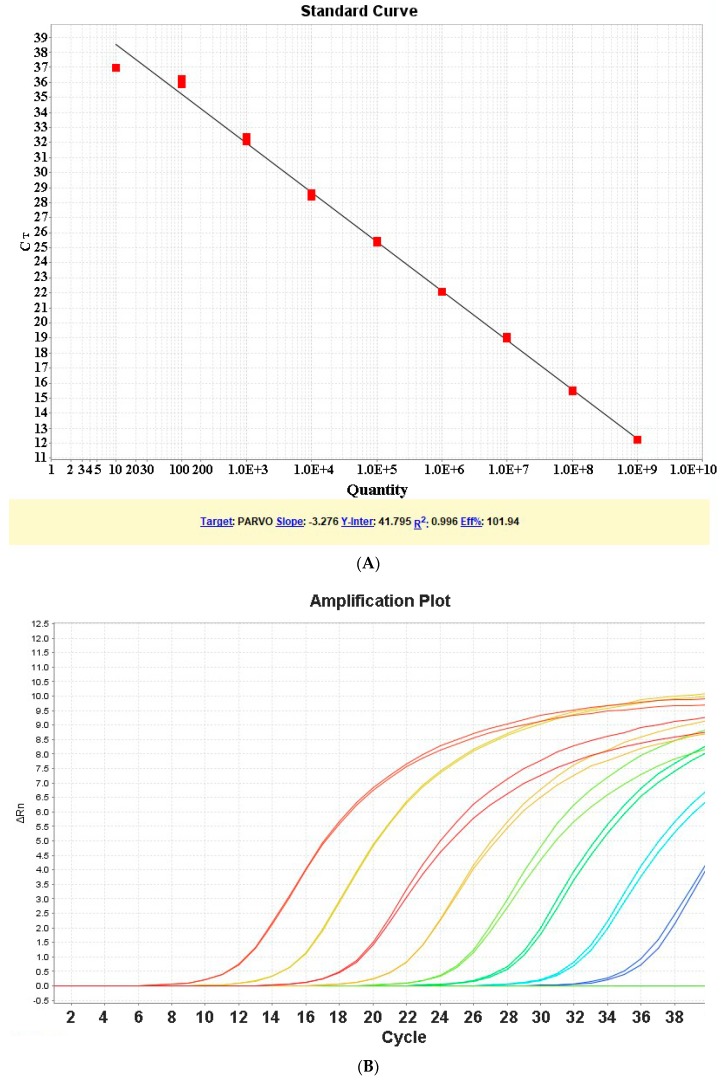

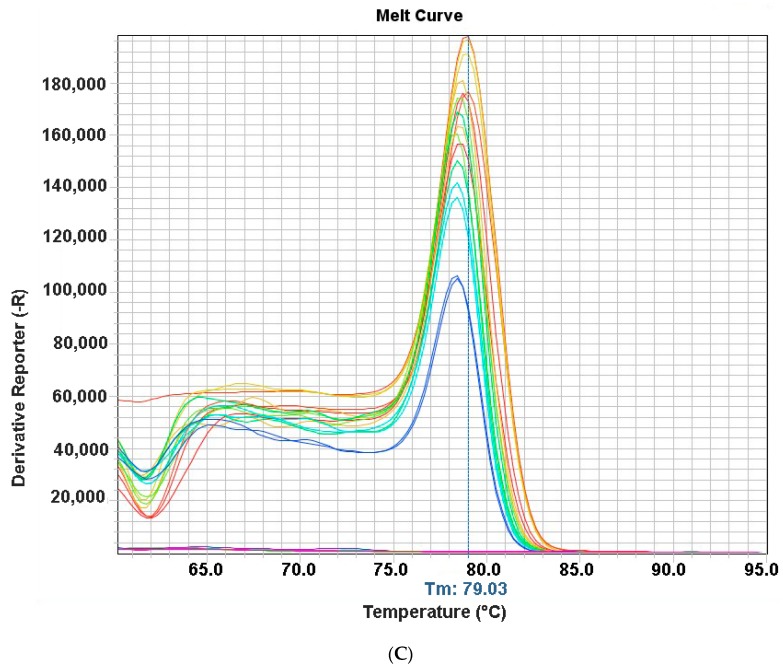
Sensitive real time fast-real time PCR (qPCR) based on SYBR^®^ Green for detection and quantification of non-structural (NS) gene of Chicken Parvovirus (ChPV)—(**A**) Standard curve using 10-fold serial dilution of NS gene plasmid of ChPV; (**B**) Amplification Plot; (**C**) Melting Curve.

The ten-fold dilutions generated a standard curve with an efficiency of 101.94% with a slope of −3.276 and a correlation coefficient of 0.996 (Figure 1).

### 3.2. Limit of Detection and Quantification

The present assay detected 10^9^ plasmid copies to five plasmid copies. The LoD was five target gene copies, and the LoQ was ten target gene copies. The melting curve was clean and with unique peaks without any alterations (Figure 1) with a melting temperature of 79.03 °C. No other peaks were observed. No curve was observed for a control that contained no template, and no dimers were observed.

### 3.3. Run Time

Each qPCR run in the FAST mode took approximately one hour in the Standard mode. If the time was increased to two and a half hours, reliable results were obtained more quickly than in the Standard mode.

### 3.4. Specificity and Sensitivity of qPCR Assay

The specificity of qPCR assay showed that the ChPV NS gene was amplified uniquely, with no amplification of DNA extracted from FAdV-1, FAdV-II, LTI and poxvirus, and the no template control, also, did not show any amplification. The sensitive qPCR assay could detect from 10^9^ plasmid copies to five plasmid copies, but at the lowest dilution, the curve did not show adequate linearity. The LoD was five target gene copies, and the LoQ was ten target gene copies. The melting curve was clean and with unique peaks without any alterations (Figure 1) with a melting temperature of 79.03 °C. No other peaks were observed. No curve was observed for a control that contained no template, and no dimers were observed.

### 3.5. Evaluation of qPCR Assay for Detection of ChPV

**Figure 2:**
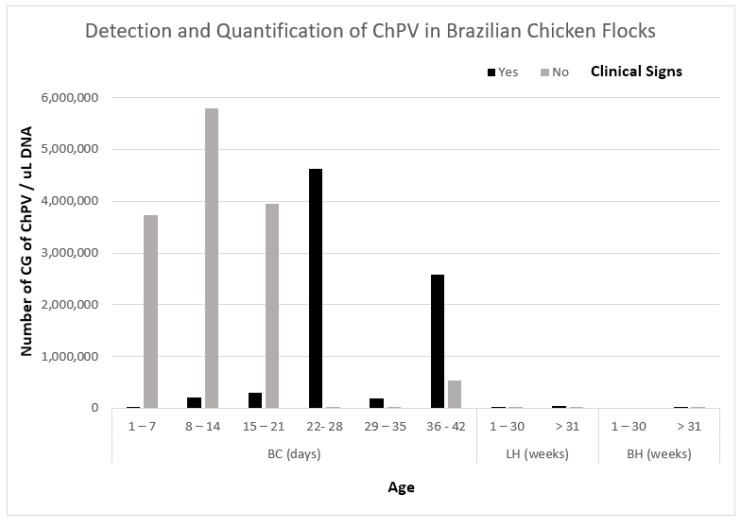
Detection and quantitation of ChPV using the sensitive fast-qPCR based on SYBR^®^ Green. ChPV = Chicken Parvovirus; BC = Broiler Chickens; LH = Layer Hens; BH = Breeder Hens; CG = Copies of Genome.

ChPV was detected in 138 out of 141 samples of enteric contents of chickens with and without the presence of enteric disease. ChPV was quantified in almost all samples. The samples contained from ten copies of the genome (CG) of ChPV to 9.6 × 10^7^ CG of ChPV. Only three samples, all from broiler chickens, were negative for ChPV. The average CG of ChPV was determined for each age range and showed varying levels of virus concentration (Table 2). The highest concentration of virus detected in samples of broiler chickens (BC) without clinical signs was 5,787,915 CG/uL DNA in an age range of 8 to 14 days old, and in BC with clinical signs was 4,631,217 in an age range of 22 to 28 days old. In breeding hens (BH), the highest viral concentration detected with clinical signs present was 4526 CG/uL DNA and in BH without clinical signs was 3294 CG/uL DNA, both in a range of ˃31 weeks. In laying hens (LH) with clinical signs, the highest concentration of virus detected was 37,321 CG/uL DNA with a range of ˃31 weeks and in the LH without clinical signs the highest concentration of virus detected was 1073 CG/uL DNA in hens in an age range of 1–30 weeks (Table 2). ChPV was quantitated in BC, LH, and BH at different ages, showing a high virus concentration in sick and healthy birds (Figure 2). ChPV was not present in any bird in the period from 1 to 30 weeks of age in LH only.

### 3.6. DNA Sequencing and Molecular Analyses

**Figure 3:**
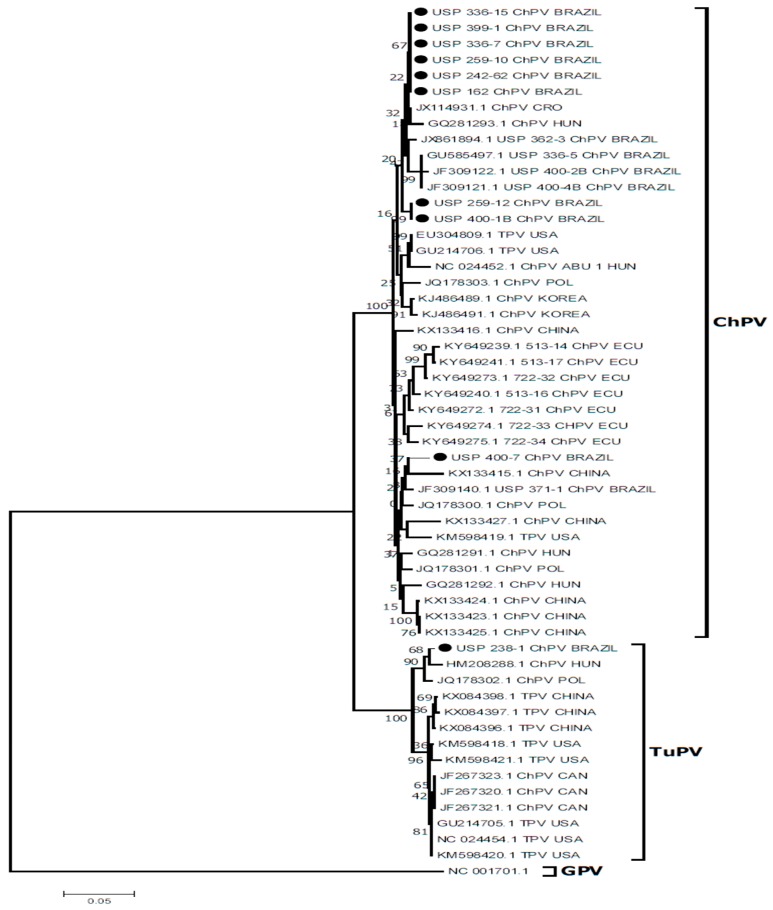
Phylogenetic relationships between the sequences of ChPV obtained here and other sequences of chicken parvovirus (ChPV) and turkey parvovirus (TuPV) from Brazil, Canada, China, Croatia, Ecuador, Hungary, Korea, Poland, and United States based on a part of NS gene nucleotide sequences. Sequences were aligned using the CLUSTAL W method in ClustalX2 2.1. The phylogenetic tree was constructed using MEGA 7 Software package. Numbers along the branches refer to bootstrap values for 1000 replicates. The scale bar represents the number of substitutions per site. Goose Parvovirus (GPV) was used as the outgroup. ● = Sequences obtained in this study; CAN = Canada; CRO = Croatia; ECU = Ecuador; POL = Poland; HUN = Hungary; USA = United States.

A phylogenetic tree showed sequences in the ChPV clustered with other sequences from Brazil, Croatia, Hungary, United States, Korea, Poland, China, and Ecuador. Only one of these sequences clustered with sequences from China and the United States. Eight of the ten sequences obtained in the present study clustered with other sequences from Brazil and formed a group with a sequence from Hungary and Croatia. One sequence obtained in the present study in the ChPV group was not grouped with most of Brazilian sequences but was aligned with another sequence from Brazil and a sequence from China in a different branch. The ChPV sequences obtained in the present study and grouped in the TuPV group were aligned with two other ChPV sequences from Hungary and Poland, and three ChPV sequences from Canada were clustered in the same group (Figure 3). The sequences obtained in the present study showed an 89.6–100% nucleotide similarity (NT) between them. The sequences obtained in the present study were compared with other ChPV sequences and showed a 97.3–99% NT similarity with other sequences from Brazil, an 89.2–95.6% NT similarity with sequences from Canada, an 88.4–98.1% NT similarity with sequences from China, an 89.4–99.7% NT similarity with the sequences from Croatia, an 88.7–97.6% NT similarity with sequences from Ecuador, an 88.3–98.8% NT similarity with sequences from Hungary, an 89.1–97.9% NT similarity with sequences from Korea, and an 88.9–98.8% NT similarity with sequences from Poland. A comparison of the sequences obtained in the present study with sequences of Turkey Parvovirus (TuPV) showed an 88.3–95.6% NT similarity with sequences from China and an 88–98.1% NT similarity with sequences from the United States.

## 4. Discussion

The present study focused on the standardization and development of a sensitive real-time fast-qPCR assay based on SYBR^®^ Green for the detection and quantification of ChPV. The diagnosis of etiological agents in poultry has been important for controlling and improving sanitation in chicken flocks [31]. Commonly, viruses, such as ChPV, have been detected using negative stain electron microscopy in which viral particles were visualized and classified according to their morphology [14,32]. Later, serological assays were used as a diagnostic tool [19], such as ELISA. Currently, several molecular diagnostic methods have been developed for the detection of ChPV, and PCR is the most employed for amplification and detection of portions of the ChPV genome [8,20]. The first PCR assay for ChPV detection focused on the NS gene and has been used in several studies; however, this assay might detect but does not quantify the virus [8]. Therefore, a qPCR for detection and quantification of ChPV has been developed for the NS gene [22] and the VP2 gene [21]. Our study showed that this real-time fast-qPCR is very sensitive, based on the SYBR^®^ Green assay, besides reducing the reaction time during amplification when compared with standard qPCR. The assay efficiency curve was 101.94% showing that the results are highly reliable, and the standard curve can be used for an absolute quantification of the viral genome. In addition, the melting curve showed a unique peak at 79.03 °C that could be used to confirm ChPV in a sample, and therefore avoid false-negative results in tissue samples with interfering DNA, such as thymus, spleen, bursa, and blood [33,34]. Hence, in the present study, ChPV was detected and quantified in the enteric contents of broilers, laying hens, and breeders using the fast-qPCR based on the SYBR^®^ Green assay that had been reported in previous studies [22]. The concentration of ChPV particles in the samples of the broiler enteric contents previously published showed a high viral concentration in healthy birds and in birds affected with an enteric virus. Similar results were found in the present study in which apparently healthy chickens also showed high viral concentrations [22]. ChPV has been detected in chickens independently of zootechnical interest and in many regions around the world with high chicken production, such as Europe [10,17,18], China [35], South Korea [36], North America [8,37,38], Brazil [9,16,24,25], and Ecuador [26,38]; however, its presence remains unknown in the rest of South America. The present study also showed that the virus was detected at earlier and later ages in chickens, demonstrating that virus dissemination can occur at any time [21]. Thus, the present study showed an average of 2.5 × 10^6^ CG in chickens with clinical signs of enteric disease, in an age range from 36 to 42 days old, and in apparently healthy animals in which a high virus concentration (CG) was detected. Presently, only three different genotypes of ChPV have been described around the world, one from the ABU 1 strain from Hungary [4,20,39] that is used as a reference genome, one in Korea [36], and one in China [35]. Therefore, the primers proposed in the present study were designed based on the alignment of the NS gene between the reference sequence of ABU 1 and the ChPV sequences from Korea and China, with a region conserved for all sequences chosen. The sequences of the NS gene obtained in the present study showed a high similarity with other ChPV sequences from other parts of the world, clustering with ChPV, and one sequence clustered with the Turkey Parvovirus (TuPV), but this result does not indicate that this sequence belongs to TuPV [28]. This result is in accordance with other studies in which some partial sequences of the ChPV NS gene cluster with the TuPV genome [28], which is useful for diagnosis but not for genetic classification for which the VP2 gene is much more suitable [28,35,36]. However, other studies should be done to determine which genotypes are currently circulating in poultry flocks in Brazil. This assay showed high sensitivity, detecting a minimum of five copies (LoD) of the viral genome and a maximum (LoQ) of 1^10^ CG of ChPV, but a different assay was not as highly sensitive [22]. Finally, the present study documented the development of a sensitive real-time fast-qPCR based on SYBR^®^ Green for detection and quantification of viral particles that could be used to determine whether a chicken flock is infected with ChPV.

## 5. Conclusions

The present study showed that the high sensitivity of the real-time fast-qPCR assay based on SYBR^®^ Green for detection and quantification of ChPV would be useful, saving execution time on screening and quantification of ChPV by almost 50% when compared with standard-qPCR.

## Figures and Tables

**Table 1:** Primers used in this study.

Primer	Gene Target	Assay	Sequences 5’–3’	Product	Reference
PVA-F	NS	qPCR	GCA ACT AAC CTG ACC GTG TG	96 bp	This Study
PVA-R			CCC GGA TTC AGA ACC AGT AT		
PVF-1		PCR	TTCTAATAACGATATCACTCAAGTTTC	561 bp	(Zsak et al., 2009)
PVR-1			TTTGCGCTTGCGGTGAAGTCTGGCTCG		

**Table 2:** Detection and quantification of Chicken Parvovirus (ChPV) in enteric contents from chickens with or without clinical signs of Runting-Stunting Syndrome (RSS).

Birds	Age		Quantitative PCR					
			Presence or Absence of Clinical Signs of Enteric Disease					
			Presence			Absence		
			Number of Positives/Total of Samples	Result of Detection	Average of CG/µL DNA	Number of Positives/Total of Samples	Result of Detection	Average of CG/µL DNA
Broilers	Days	1–7	8/9	+	1368	5/5	+	3,722,751
		8–14	8/8	+	215,461	4/4	+	5,787,915
		15–21	16/18	+	300,689	3/3	+	3,943,261
		22–28	21/21	+	4,631,217	2/2	+	202
		29–35	13/13	+	190,928	2/2	+	24,621
		36–42	25/25	+	2,586,163	8/8	+	543,367
Layer hens	Weeks	1–30	6/6	+	1148	5/5	+	1073
		>31	4/4	+	37,321	1/1	+	10
Breeder Hens		1–30	0/0		Na	0/0		Na
		>31	4/4	+	4526	3/3	+	3294

CG = Copies of genome; ChPV = Chicken parvovirus; Na = does not apply.
